# Efficacy of Low-Dose Radiotherapy for Pain Control in Elderly Patients With Advanced Knee Osteoarthritis: A Prospective Single-Arm Study From India

**DOI:** 10.7759/cureus.115078

**Published:** 2026-08-24

**Authors:** Amiya Gopal Ranja, Suparna K Pal, Chandrima Banerjee, Pratyusha Mukherjee, Rajesh Pramanik, Aloke Ghosh Dastidar

**Affiliations:** 1 Radiation Oncology, Institute of Post Graduate Medical Education and Research, Kolkata, IND; 2 Physical Medicine and Rehabilitation, Institute of Post Graduate Medical Education and Research, Kolkata, IND

**Keywords:** india, knee osteoarthritis, low-dose radiotherapy, pain-control, radiotherapy in benign disease

## Abstract

Background

Osteoarthritis is one of the commonly occurring causes of disability and pain in the elderly Indian population. Low-dose radiotherapy (LDRT) has been used in Europe and the US for symptomatic management.

Objectives

To evaluate the efficacy of LDRT in achieving clinically significant pain reduction (≥30% improvement in Visual Analogue Scale (VAS)), its durability over 12 months, and its safety in elderly patients with advanced knee osteoarthritis who were unsuitable for or declined surgical intervention.

Methods and materials

One hundred and six patients aged >50 years with Kellgren-Lawrence grade III-IV knee osteoarthritis (OA) received LDRT using cobalt-60, delivering 3 Gy in six fractions (0.5 Gy/fraction, two times a week). Pain was assessed using VAS at baseline and at four weeks, four months, eight months, and 12 months. Inflammatory biomarkers (erythrocyte sedimentation rate (ESR) and C-reactive protein (CRP)) and treatment-related toxicity were also evaluated.

Results

LDRT produced a significant reduction in pain compared with baseline, with mean VAS decreasing from 7.9±0.6 to 3.5±1.2 at 4 weeks and remaining significantly improved through 12 months (4.0±0.9; all P<0.001). Overall, 88 subjects out of 90 (97.8% of evaluable subjects) achieved at least a 30% reduction in pain. CRP and ESR levels both decreased significantly after treatment (P=0.002 and 0.006, respectively). No grade ≥3 adverse events, radiation-induced tissue injury, or unexpected toxicities were observed.

Conclusions

LDRT was safe, well tolerated, and provided reasonable pain relief for up to 12 months in elderly patients with advanced knee OA, subject to limitations of this study. These findings support further randomized sham-controlled trials incorporating functional outcomes, quality-of-life measures, and translational biomarkers to define the role of LDRT in contemporary osteoarthritis management.

## Introduction

Radiotherapy (RT) has been used to treat benign conditions over the decades [1]. RT has different biologic effects at different doses. Conventional and hypofractionated schedules show antiproliferative principles that are used to treat malignant diseases. At doses less than 1 Gray (Gy) per fraction, radiotherapy is known to show strong anti-inflammatory effects [2]. Low dose radiation therapy (LDRT) has been successfully used to treat painful musculoskeletal conditions by some authors [1]. Conditions such as trochanter bursitis, medial and lateral epicondylitis, plantar fasciitis, and osteoarthritis (OA) of both large and small joints have benefited from LDRT [3].

OA of big joints is one of the leading causes of morbidity in the elderly population in India [4]. The prevalence of knee OA in India for individuals above 50 years old was estimated to be around 27-39%, with a significant increase in prevalence among women and with increasing age, with some studies reporting rates as high as 64.3% in the elderly population of rural areas in India [5]. It not only causes pain and limits mobility, but also leads to loss of employment, poor quality of life, and a huge caregiver burden. Conservative management of OA includes analgesics, chiropractic, and physiotherapy, which have been the mainstay of management for OA [6]. The alternative pathway has been joint replacement, which is a costly and high-risk invasive procedure. Moreover, multiple joint replacements may not be a suitable option for patients who successively develop OA of multiple joints.

Although LDRT has been used for decades in Europe for painful musculoskeletal conditions, high-quality randomized evidence remains mixed, and recent meta-analyses have questioned its efficacy. Data from low- and middle-income countries, including India, are scarce. We therefore conducted a prospective single-arm study to evaluate the efficacy, safety, and durability of LDRT for pain relief in elderly Indian patients with advanced knee OA who were unsuitable for or declined surgery.

## Materials and methods

Objectives

Primary Objective

The study aims to determine the proportion of patients achieving a clinically significant reduction in pain, defined as ≥30% improvement from baseline on the Visual Analogue Scale (VAS), at four weeks after completion of LDRT.

Secondary Objectives

The study aims to evaluate the durability of pain relief at four, eight, and 12 months following LDRT, assess the safety and tolerability of LDRT, including both acute and late treatment‑related toxicity up to 12 months, and examine changes in systemic inflammatory markers such as erythrocyte sedimentation rate (ESR) and C-reactive protein (CRP) in response to LDRT.

Sample size

The study was designed to be considered successful if there is at least a 30% reduction in pain severity as per the VAS in around 60% of OA patients being treated, with a margin of error of 10%. 

The sample size was calculated using the standard formula for estimating a population proportion:

\(\begin{equation} n=\frac{4PQ}{d^{2}} \end{equation}\)

where n is the required sample size, P is the anticipated proportion, Q is (1-P), and d is the desired absolute precision.

Using P = 0.60, Q = 0.40, and d = 0.10, the sample size was calculated as follows:

\(\begin{align} n &= \frac{4 \times 0.60 \times 0.40}{(0.10)^2} \\ &= \frac{0.96}{0.01} \\ &= 96 \end{align}\)

To account for an anticipated attrition rate of 10%, the adjusted final sample size was kept at 106.

Participants

The identified patients fulfilled all the inclusion criteria. The inclusion criteria were age >50 years; knee OA of Kellgren-Lawrence grade 3 or 4; pain, disability, or deterioration in quality of life that was inadequately controlled with exercise, topical analgesics, and a single nonsteroidal anti-inflammatory drug (NSAID); and either being unfit for joint replacement because of comorbidities or having refused surgery for any reason.

Patients with a history of irradiation in the past at or near the affected joints, uncontrolled comorbid conditions, those with BMI >39 Kg/m2, history of collagen vascular disease, fibromyalgia, or patients participating in other degenerative arthritis clinical trials were excluded.

Intervention

Our patients were all treated with a Cobalt-60 beam (1.25 MV average) from a Bhabatron II machine (an Indian make Cobalt-60 machine from Panacea Medical Technologies Pvt. Ltd., Bengaluru, Karnataka, India). They were treated with anterior-posterior and posterior-anterior (AP-PA) fields, using the SSD technique (80 cm SSD), at the dose of 3Gy in 6 fractions once daily, two times a week, prescribed to the midplane. This dose was chosen as it was the most common dose used in knee OA [1].

**Figure 1:**
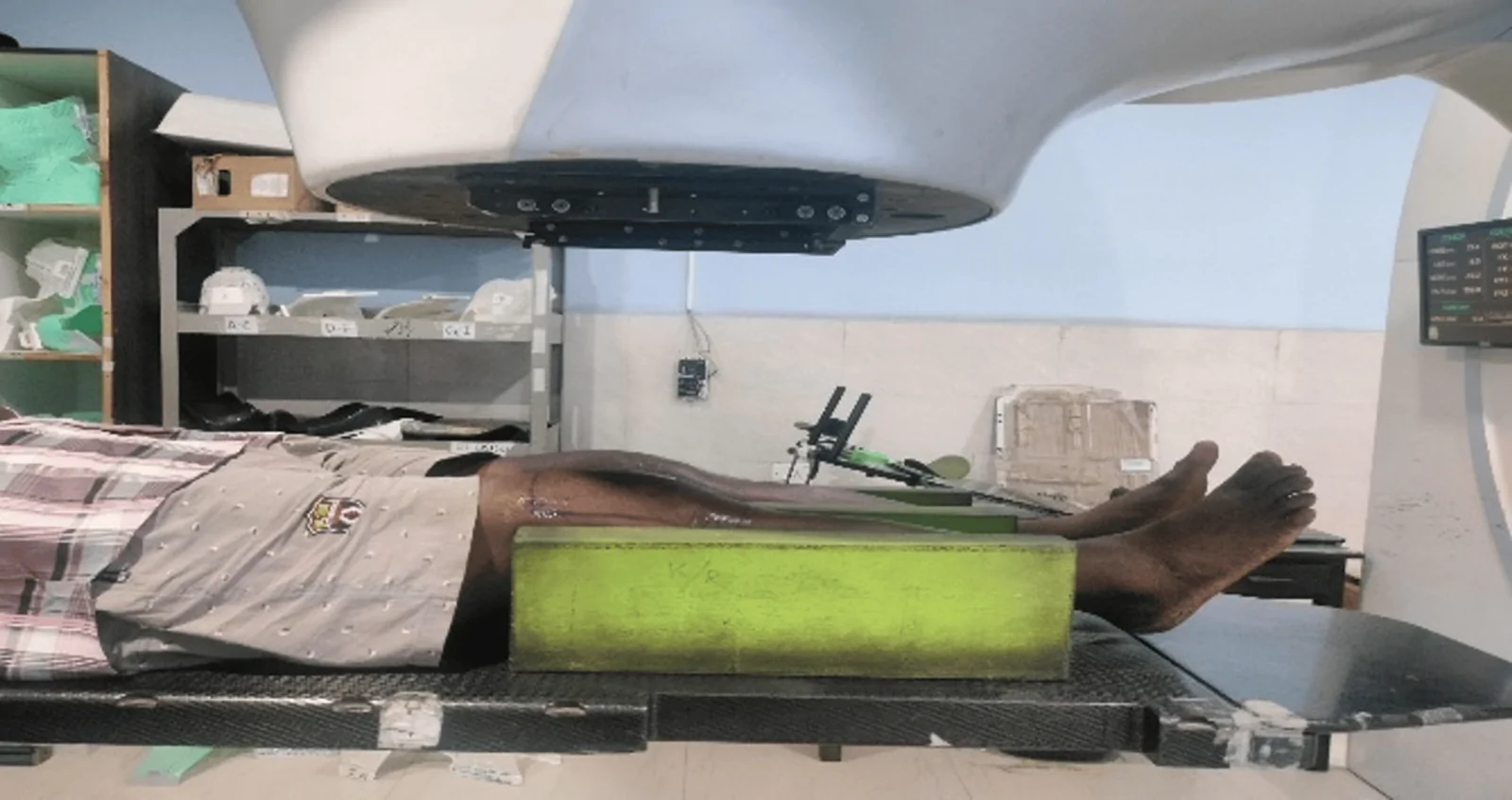
Execution of the treatment at a Cobalt-60 machine with knee rest.

The fields were defined based on recommendations as mentioned by Dove et al. [1]. The superior border was positioned 8 cm superior to the joint space, the inferior border 8 cm inferior to the joint space, the medial border 3 cm medial to the medial femoral condyle, and the lateral border 3 cm lateral to the lateral tibial condyle. Two-dimensional conventional planning was done, and immobilization was used as deemed appropriate with knee-first supine posture. Daily setup was based on Skin markings since EPID (Electronic Portal Imaging Device) was not available with Bhabatron II (Figure 1).

Outcome measurement

Pain score was calculated using the VAS. It was measured at baseline (pre-LDRT) and at follow-up visits at four weeks, four months, eight months, and 12 months after the end of LDRT in person by a blinded assessor (staff nurse).

Blood reports for complete blood count (CBC), liver function test (LFT), urea, creatinine, glucose, total protein, albumin, ESR, and CRP were collected at screening, four-week follow-up, four-month follow-up, and 12-month follow-up after the end of LDRT.

Digital X-ray was done during pre-LDRT, at four months, and at 12 months after LDRT.

Patients were advised to take analgesics as required (Tab. paracetamol 15 mg/kg per dose, not more than 3G/day) and to do exercises, and were asked about changes in dosage and the number of other rescue medications taken (Tab. diclofenac 50 mg stat dose).

Weekly check-ups were performed during radiotherapy to evaluate acute toxicities according to the Common Terminology Criteria for Adverse Events, version 5.0 (CTCAE v5.0) [7].

Ethical approval and trial registration

The study was approved by the Institutional Ethics Committee, Institute of Post Graduate Medical Education and Research (IPGME&R), Kolkata (approval no. IPGME&R/IEC/2023/474; dated June 10, 2023). The trial was registered with the Clinical Trials Registry - India (CTRI; registration no. CTRI/2024/11/076610).

Statistical analysis

The collected data were analyzed and presented in the form of frequency tables for descriptive statistics. In case of pre-LDRT and post-LDRT comparison for continuous data, a paired t-test was used.

In case of pre-LDRT and post-LDRT comparison where the data were ordinal, a non-parametric Wilcoxon test was used.

In case of loss to follow-up for any reason, despite best efforts, the missing participant was included in the analysis only up to the time point at which the participant's data were available.

Longitudinal changes in VAS scores were evaluated using a linear mixed-effects model with time treated as a categorical fixed effect and patient-specific random intercepts to account for within-patient correlation.

## Results

Among 106 patients who started, 16 did not complete the planned intervention. All 16 patients were contacted telephonically. Each subject was tried at least three times, with a gap of at least two days and at different hours, or till they or their family members could be contacted. Twelve subjects could be contacted, but four could not be.

Out of the 12 subjects, six could not come as their primary caregiver was not available. Two were “feeling better” but did not want to travel.

**Figure 2:**
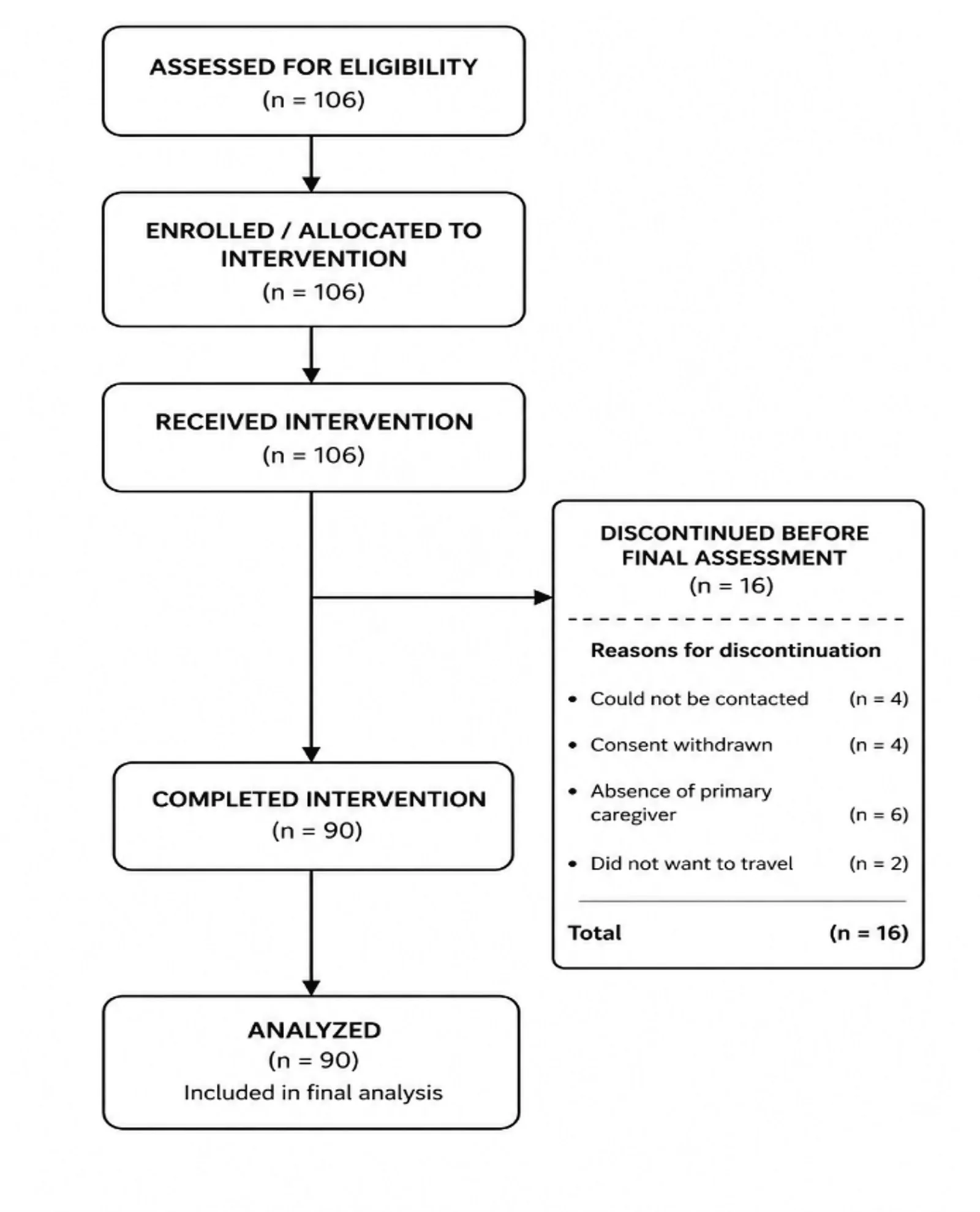
CONSORT diagram. CONSORT, Consolidated Standards of Reporting Trials.

Four of the subjects did not wish to take radiation anymore, as they felt it might have side effects. The potential risks and benefits of continuing radiotherapy were explained to them again; however, they subsequently withdrew their consent and were recorded as consent withdrawals (Figure 2). The study therefore lost nearly 15% of the patients during this course of time and is therefore likely to have potential attrition bias.

While all the subjects were included in the demographic analysis, only 90 were included in the outcome analysis. The demographic and disease characteristics are shown in Table 1.

**Table 1:** Baseline characteristics of osteoarthritis (OA) patients (N=106).

Characteristic	Category	Male (n=42)	Female (n=64)	Total n (%)
Age group (years)	50-60	10	44	54 (50.95)
	61-70	28	20	48 (45.28)
	>70	4	0	4 (3.77)
Residence	Urban	14	26	40 (37.74)
	Rural	28	38	66 (62.26)
Occupation	Homemaker	0	30	30 (28.31)
	Shopkeeper	8	14	22 (20.75)
	Farmer	14	4	18 (16.98)
	Laborer	6	4	10 (9.43)
	Carpenter	8	0	8 (7.55)
	Business	4	2	6 (5.66)
	Gardener	2	4	6 (5.66)
	Fisher	0	6	6 (5.66)
Latrine type	Western	10	24	34 (32.08)
	Indian	32	40	72 (67.92)
OA grade	Grade 3	28	24	52 (49.06)
	Grade 4	14	40	54 (50.94)
Sex distribution	Male	42	-	42 (39.62)
	Female	-	64	64 (60.38)

VAS scores at each follow-up were compared with baseline using the Wilcoxon signed-rank test. All comparisons were statistically significant (P < 0.001) (Table 2). 

**Table 2:** Comparison of VAS scores following treatment with baseline using the Wilcoxon signed-rank test. Values are presented as mean ± standard deviation (SD) and median (range). Comparisons between baseline and each follow-up visit were performed using the Wilcoxon signed-rank test for paired observations. The test statistic is reported as the standardized Z value, and corresponding two-tailed P values are presented. *P < 0.05 indicates statistical significance. N represents the number of patients with paired baseline and follow-up observations available for analysis at each time point. VAS, Visual Analogue Scale.

Time point	N	Mean ± SD	Median (range)	Wilcoxon signed-rank test (Z)	P value
Baseline	106	7.90 ± 0.58	8 (2)	-	-
4 weeks	90	3.50 ± 1.20	3 (4)	-5.902	<0.001*
4 months	84	3.70 ± 0.86	4 (4)	-5.771	<0.001*
8 months	72	4.10 ± 0.99	4 (4)	-5.681	<0.001*
12 months	68	4.00 ± 0.85	4 (3)	-5.702	<0.001*

Longitudinal changes in VAS scores were evaluated using a linear mixed-effects model with time as a categorical fixed effect and patient-specific random intercepts. The analysis demonstrated a statistically significant effect of time on VAS scores (p < 0.001). VAS decreased markedly from baseline (7.92 ± 0.58) to four weeks (3.53 ± 1.17), with a modest increase at four months (3.71 ± 0.86), eight months (4.10 ± 0.99), and 12 months (4.00 ± 0.85), while remaining substantially below baseline. These findings were consistent with generalized estimating equations using an exchangeable working correlation structure and repeated-measures ANOVA among patients with complete observations.

On paired t-test analysis of the inflammatory biomarkers, both ESR and CRP levels were found to be significantly reduced after LDRT, suggesting a reduction in disease activity following the intervention (Table 3).

**Table 3:** Changes in inflammatory biomarkers following LDRT. Values are presented as mean ± standard deviation (SD). Mean difference represents the paired difference (before treatment − after treatment). Statistical comparisons between pre- and post-treatment measurements were performed using a two-sided paired Student's t-test. The 95% confidence interval (CI) is reported for the mean paired difference. Statistical significance was defined as P < 0.05. ESR, erythrocyte sedimentation rate; CRP, C-reactive protein; LDRT, low-dose radiation therapy; t, paired t-test statistic; df, degrees of freedom.

Biomarker	Before treatment (Mean ± SD)	After treatment (Mean ± SD)	Mean difference	95% CI of mean difference	t (df)	Two-sided P value
ESR (mm/hr)	34.00 ± 14.62	31.96 ± 14.12	2.04	0.60 to 3.48	2.822 (89)	0.006
CRP (mg/dL)	3.05 ± 2.94	2.44 ± 2.57	0.62	0.23 to 1.01	3.148 (89)	0.002

The recorded use of analgesics showed no increase in frequency of the same. No patients required more than two rescue medications of Tab. diclofenac 50 mg at any given week. No toxicity was noted either during treatment or follow-up visits.

## Discussion

OA is a chronic degenerative joint disease that affects a significant portion of the elderly population. It not only causes pain and disability but also imposes a substantial socioeconomic burden. While conventional treatment modalities such as analgesics, physical therapy, intra-articular injections, and surgery remain the mainstay of OA management, their limitations necessitate the exploration of alternative therapies.

LDRT has historically been used for inflammatory and degenerative conditions in Western countries [1]. Most of the recommendations on treatment of OA do not consider LDRT as a standard of care [6]. The German Society for Radiation Oncology guidelines recommend the use of LDRT in hand OA but are silent on knee OA [8].

The use of radiotherapy in OA remains underexplored in India. This study was conducted to evaluate the efficacy of LDRT in reducing pain and improving the quality of life in elderly OA patients, thereby addressing a crucial treatment gap.

In the present study, out of the recruited 106 subjects, 16 subjects defaulted, and we analyzed 90 patients. To the best of our knowledge, this is one of the largest prospectively collected single-arm datasets from an Indian population.

In our study population, 54 patients (50.95%) were in the age group of 50-60 years, and 20 patients (45.28%) were in the age group of 61-70 years. Desai et al. (2021) showed that 57.93% of OA patients were in the age group of 61-70 years [5]. Among the study subjects, 64 patients (60% ) were female. Desai et al. (2021) found a female preponderance (64.63% vs. 35.37%) among osteoarthritis patients [5]. Among the study subjects, more than 66 patients (62%) were from a rural background. Sharma et al.(2013) had shown a significant difference in the prevalence of OA between rural (56.6%) and urban areas (32.6%) [9]. Almost half of the female patients (30 out of 64) were homemakers (46.88%), whereas one-third of the male patients (14 out of 42) were farmers.

In our study population, more than 72 patients (67%) used Indian-type latrines. But Pal et al. (2016) showed prevalence of OA was found to be significantly higher (p = 0.001) in patients who used Western-type latrines (42.1%) as compared to those who used Indian-type latrines (29.7%) or both types (38.8%) [10]. Being a state-run medical college, catering to the most marginalized population, this may be due to the presentation bias of our catered population.

The present study used a two-dimensional technique, as used in most of the studies. However, recently three-dimensional (3DCRT) planning has been successfully used by some authors [11]. Recently, Steike et al. (2026) from the International Benign Target Volume Group have published a consensus statement for 3DCRT target volume delineation [12].

Pain relief was the primary objective of this study, and it was assessed using the VAS. The results demonstrated a statistically significant reduction in pain scores, confirming the effectiveness of LDRT on a sustained basis till 12 months of follow-up.

The Wilcoxon Signed-Rank Test confirmed a highly significant improvement (p<0.001) at all follow-up points. Notably, 97.78% of patients (88 out of 90 evaluable patients) showed at least a 30% improvement in pain scores, reinforcing LDRT’s efficacy, though none of our subjects had complete relief (VAS 0).

Donaubauer et al. (2020) showed pain improvement in 69% of patients [12]. Of these, 28% reported complete pain relief, while 41% experienced partial pain reduction [13]. Hautmann et al. (2020) conducted an analysis of 295 osteoarthritic joints, of which the pain improvement rate was 81%, with 35% of patients reporting complete pain relief and 46% experiencing partial pain reduction [14]. Hautmann et al. (2020) conducted a study evaluating radiotherapy for osteoarthritis in 66 ankle and tarsal joints, which showed an overall pain improvement rate of 85%, with 39% of patients reporting complete pain relief and 46% experiencing partial pain reduction [15]. 

Thus, in our study group, significant pain relief was found in nearly all the subjects, though the maximum reported relief was limited to a 75% reduction in VAS.

Ruhle et al. analyzed data from 1185 radiotherapy sites of 970 patients from multiple centres and showed that 66% of the patients had good control irrespective of age [16]. Alvarez et al. (2022) analyzed the effects of LDRT on hand osteoarthritis, reporting that 62.5% of patients experienced significant pain relief [17].

Hammadeh et al. (2025) published a systematic review and meta-analysis, which did not find any impact of LDRT on pain control in osteoarthritis [18]. The meta-analysis initially included and found 12 studies fit for the meta-analysis but subsequently used only six studies for the same. The reason for excluding those six studies was not mentioned in the meta-analysis. The final pain outcome was available in only four of the studies (Mahler et al., 2019; Fazliat-Panah et al., 2025; Niewald et al., 2022; Minten et al., 2018) [19-22]. The included negative Arthro-Rad trial results had only three months of follow-up [23]. The 12-month follow-up publication published in 2024 was not included due to concerns about a high risk of bias due to missing data [23]. Moreover, the study by Minten et al. included patients with only hand osteoarthritis [22].

**Table 4:** Comparison of studies exploring low-dose radiotherapy for OA. LDRT, low-dose radiotherapy; OA, osteoarthritis; RT, radiotherapy; Gy, Gray.

Study	Joint/site	Design	Radiotherapy technique	Dose/fractionation	Sham/control	Earliest prespecified/reported assessment of pain response	Placebo/sham response	Overall interpretation
Minten et al. (2018) [22]	Hand OA	Randomized, blinded, sham-controlled	External-beam low-dose RT to symptomatic hand joints	6 Gy in 6 × 1 Gy	Sham irradiation	Primary endpoint at 3 months; pain/symptom outcomes assessed during follow-up	No significant difference between RT and sham	Strong evidence that symptomatic improvement may be substantially influenced by placebo/contextual effects
Mahler et al. (2019) [19]	Knee OA	Randomized, double-blind, sham-controlled	External-beam LDRT	6 Gy in 6 × 1 Gy	Sham RT	3 months primary endpoint; further follow-up to 12 months	44% RT vs 43% sham responders	Very strong demonstration of a large sham/placebo response
Niewald et al./ArthroRad (2022) [21]	Knee and hand OA	Randomized, single-blind dose-comparison	External-beam LDRT	3 Gy/6 × 0.5 Gy vs 0.3 Gy/6 × 0.05 Gy	No true sham	Improvement assessed at 3 months	Not applicable	Both radiation doses produced improvement; biological RT effect cannot be separated from placebo/regression to the mean
Niewald et al./ArthroRad (2024) [23]	Knee and hand OA	Randomized, single-blind dose-comparison	External-beam LDRT	3 Gy/6 × 0.5 Gy vs 0.3 Gy/6 × 0.05 Gy	No true sham	Longer-term assessment to 12 months	Not applicable	Both radiation doses produced improvement; biological RT effect cannot be separated from placebo/regression to the mean
Fazilat-Panah et al. (2025) [20]	Knee OA	Randomized, double-blind, sham-controlled	External-beam LDRT	3 Gy/6 × 0.5 Gy	Sham RT	Pain/function assessed at approximately 1, 3, and 6 months	Active RT produced greater improvement than sham	Provides evidence for a treatment-specific effect beyond placebo
Present study (2026)	Knee OA	Single-arm open-label	External-beam LDRT	3 Gy in 6 fractions (0.5 Gy per fraction)	None	Pain assessed at 4 weeks, 4, 8, and 12 months	Not applicable	Evidence for the need for a sham-controlled trial in the Indian population

Although the reductions in inflammatory markers were modest in our study, they align with the hypothesis that LDRT modulates inflammation locally rather than inducing broad systemic changes. The interim analysis of the IMMO-LDRT01 trial, by Donaubauer et al. (2021), noted immunomodulatory effects, with a reduction in pro-inflammatory cytokines and an increase in anti-inflammatory markers in peripheral blood [24].

Safety and tolerability of LDRT

Our study also assessed the safety profile of LDRT. There were no severe adverse effects, and no radiation-induced tissue damage was observed. Given its tolerability, LDRT appears to be a suitable therapeutic option for elderly patients, particularly those at risk from pharmacological treatments or surgery. The meta-analysis by Hammadeh et al. (2025) did not find any significant toxicity with the use of LDRT for OA when compared to sham treatment [18].

Strengths

This research provides evidence supporting LDRT for OA in an Indian population in a prospective design. Unlike retrospective studies, this study followed patients over 12 months, allowing for an extended assessment of treatment efficacy.

Limitations

The absence of a sham-control arm and the progressive loss to follow-up limit the strength of causal conclusions. Since some Western studies [19,22] found a significant placebo effect in their sham-controlled study design, it is not possible to say how far the present results have been influenced by the placebo effect. Functional outcomes (e.g., mobility, daily activities such as the Western Ontario and McMaster Universities Osteoarthritis Index (WOMAC), Knee Injury and Osteoarthritis Outcome Score (KOOS), or quality-of-life instruments) and patient-reported quality-of-life measures were not collected, restricting the clinical interpretability of the observed pain reduction [25,26].

Other inflammatory markers (e.g., IL-1Beta, TNF-alpha, IL-6) have not been measured in our study. For future studies, these markers are to be measured quantitatively before and after treatment to validate the study more efficiently.

The proposed two-stage Knee Osteoarthrosis Radiotherapy Trial (KORT) study using a multidisciplinary pathway with sham treatment is expected to provide a clearer idea about the role of LDRT in Knee OA in the German population [27].

## Conclusions

In this prospective single-arm study, low-dose radiotherapy was safe and associated with significant, durable reductions in pain scores for up to 12 months in elderly Indian patients with advanced knee osteoarthritis. These results support further evaluation in randomized, sham-controlled trials that include functional and quality-of-life endpoints.
